# Single-cell sequencing analysis reveals the relationship between tumor microenvironment cells and oxidative stress in breast cancer bone metastases

**DOI:** 10.18632/aging.204885

**Published:** 2023-07-19

**Authors:** Minmin Zhang, Xiao Chai, Li Wang, Ke Mo, Wenyang Chen, Xiangtao Xie

**Affiliations:** 1 Department of Breast and Thyroid Surgery, Liuzhou People’s Hospital, Liuzhou 545006, Guangxi, People’s Republic of China; 2 Biology Institute, Guangxi Academy of Sciences, Nanning 530007, Guangxi, People’s Republic of China; 3 Department of Orthopedics, Liuzhou People’s Hospital, Liuzhou 545006, Guangxi, People’s Republic of China; 4 Department of Orthopedics, The Fourth Affiliated Hospital of Guangxi Medical University, Liuzhou 545005, Guangxi, People’s Republic of China; 5 Department of Orthopedics, Liuzhou Worker’s Hospital, Liuzhou 545005, Guangxi, People’s Republic of China

**Keywords:** breast cancer bone metastasis, oxidative stress, apoptosis, bone remodeling, single cell RNA sequencing

## Abstract

Bone metastasis (BM) is one of the main manifestations of advanced breast cancer (BC), causing complications such as pathological fractures, which seriously affects the quality of life of patients and even leads to death. In our study, a global single-cell landscape of the tumor microenvironment was constructed using single cell RNA sequencing data from BM. BC cells were found to be reduced in the BM, while mesenchymal stem cells (MSCs), Fibroblasts and other cells were significantly more abundant in the BM. The subpopulations of these cells were further identified, and the pathways, developmental trajectories and transcriptional regulation of different subpopulations were discussed. The results suggest that with the development of BM, BC cells were vulnerable to oxidative damage, showing a high level of oxidative stress, which played a key role in cell apoptosis. Fibroblasts were obviously involved in the biological processes (BPs) related to ossification and bone remodeling, and play an important role in tumor cell inoculation to bone marrow and growth. MSC subpopulations were significantly enriched in a number of BPs associated with bone growth and development and oxidative stress and may serve as key components of BC cells homing and adhesion to the ecological niche of BM. In conclusion, our research results describe the appearance of tumor microenvironment cell subpopulations in breast cancer patients, reveal the important role of some cells in the balance of BM bone remodeling and the imbalance of BM development, and provide potential therapeutic targets for BM.

## INTRODUCTION

Breast cancer (BC) is the most common cancer in women worldwide and the leading cause of cancer death in women. The ER-positive (ER+) breast cancer is the most common subtype of breast cancer, accounting for 68% of all breast cancer types. Because of its heterogeneous nature, it is particularly difficult to diagnose and evaluate clinically, and therefore the incidence and mortality rates are on the rise and the disease burden is increasing [1]. BC patients may experience bone metastasis (BM), which is currently considered incurable [2]. About 70% or more of advanced breast cancer will develop distant metastases, and about 83% of them have bone as the first metastatic site. Once bone metastases occur, osteolytic destruction is formed, which is prone to fracture and nerve compression in late stage, and obvious pain and paralysis symptoms, which seriously affects patients’ survival quality and survival rate [3]. According to the World Health Organization’s International Agency for Research on Cancer (IARC) 2022 cancer statistics report, breast cancer accounts for the first place among all cancers in women with 31% of new cases and the second place among all cancer types in women with 15% of mortality. However, because the molecular mechanisms of BM have not been fully elucidated, the efficacy of existing treatments is limited and they have failed to significantly improve the overall survival rate of patients [1, 4, 5].

BM is a complex multi-step process, including the disruption of the dynamic balance of bone remodeling, vascularization in the tumor, regulation of immune cells such as bone marrow mesenchymal stem cells (BMSCs), adipocytes and macrophages, etc. The molecular mechanisms are very complex, and these cell types and their secreted factors together constitute the tumor microenvironment (TME). These cell types and their secreted factors together constitute the TME, and are closely related to the occurrence of breast cancer bone metastasis [6, 7]. The complex composition of the TME includes fibroblasts, immune cells, adipocytes, vascular endothelial cells and extracellular matrix, etc. The complex molecular components and cellular changes in the TME are essential for promoting cancer metastasis [8]. Previous studies have shown that relevant fibroblasts can promote the metastasis of breast cancer cells. These fibroblasts play a key role in the bone colonization of breast cancer cells by influencing the intrinsic tumor characteristics and TME [9]. In addition, MSC localized in breast cancer form “tumor xenografts” with tumor cells, leading to tumor cell growth and bone metastasis [10]. In addition, patients with breast cancer may induce oxidative reactions in the body through a variety of pathways, placing the body in a state of oxidative stress and adversely affecting the prognosis of the patient [11]. The ability of breast cancer to metastasize is closely related to the redox status of cells [12]. However, the ecology of specific cells in BM is still unknown to a large extent. Therefore, further research on the cell level in BM is of great scientific significance and is of great significance for developing new strategies for BM treatment.

In this study, we used single cell technology to construct a global single cell landscape atlas of BM, comprehensively discussed the ecosystem of BM microenvironment, and revealed the imbalance of BM bone remodeling balance and the important role of some cells in BM development.

## MATERIALS AND METHODS

### Data collection and processing

BM-related single cell RNA sequencing (scRNA-seq) data were obtained from Gene Expression Omnibus (GEO, https://www.ncbi.nlm.nih.gov/geo/), dataset GSE190772 based on the GPL24676 platform, including BM of two bilateral bone metastases collected in a patient initially diagnosed with ER+ primary breast cancer. In addition, dataset GSE131007 based on the GPL20301 and GPL24676 platforms, including three primary tumor tissues from mouse xenografts of human BC patient origin and one BM tissue. Among them, cells from murine cells or human-murine doublets were excluded. Total 3 BM tissues and 3 primary samples, and the primary samples were the control samples in this study.

### Construction of single cell atlas

Single-cell data were merged using the IntegrateData function [13] of the Seurat package [14] in R language, and cell clustering analysis was performed according to default parameters, filtering for cells with top and bottom 1% gene count and >10% mitochondrial content. The clustering results were downscaled and visualized [15] based on a uniform manifold approximation and projection (UMAP) for dimension reduction technique and projected onto a two-dimensional image defined as a single-cell atlas. In addition, cell types were annotated according to cell markers known from previous studies [16].

### Differential gene expression analysis

The differentially expressed genes (DEGs) in each cluster between single cells of primary tumor tissue and BM tissues were identified using the “FindAllMarkers” function, and differences with a adjusted P-values < 0.05 and |log fold change (logFC)| > 0.5 were considered significant.

### Functional enrichment and gene enrichment analysis

To further explore the biological processes and pathways involved with genes that showed dysregulated expression in different cell clusters, Gene Ontology (GO) terminology and Kyoto Encyclopedia of Genes and Genomes (KEGG) enrichment analyses were performed based on the expression of marker genes. The R package clusterProfiler [17] for enrichment analysis regarding biological processes (BPs) of GO and KEGG signaling pathways, and P<0.05 were considered significant.

### Cellular scoring of oxidative stress-related gene sets

The AddModuleScore function [18] in the Seurat package was used to score oxidative stress-related pathways.

### Pseudo-time analysis

The differentiation developmental trajectory of dysregulated cells in primary tumor tissues and BM tissues was reconstructed using the Monocle 3 package [19] in R language and visualized by UMAP. Subsequently, the cells were sorted according to their progression through the developmental program.

### Gene regulatory network (GRN) analysis

In addition, using the Python module tool pySCENIC [20], this study comprehensively reconstructed the transcription factor-centered gene regulatory network to further explore the regulatory mechanisms of dysregulated cells.

The workflow started with describing the input single-cell expression level profile matrix, and then using a regression method for each target (GRNBoost2) to infer co-expression modules. The results allowed us to determine which indirect targets were trimmed based on the discovery of cis-regulatory patterns (cisTarget). Subsequently, AUcell was used to quantify the activity of those regulators by enriching and scoring the regulator target genes to obtain a regulon activity score (RAS). The single-cell data were further downscaled using the RAS matrix and a regulon specificity score (RSS) was calculated based on the Jensen-Shannon divergence (JS scatter) and used to identify regulators specific for certain cell populations. The most specific and significant regulons were mapped to single cell cluster profiles and validated using massively parallel sample sequencing (SEEK database). Finally, a connection specificity index (CSI) matrix was calculated, and the regulators were hierarchically clustered according to CSI to define regulator modules that could be used to identify relationships between regulator modules and regulators. Those relationships were then visualized using the R package ComplexHeatmap.

### Cellular communication

Signal transduction emphasizes the manner and outcome of signal reception and the signal conversion after reception, with ligand-receptor binding being one of the main forms of signal transduction between neighboring cells. In this study, high confidence ligand-receptor interactions between subpopulations of cells were identified by the R language package iTALK. It preferentially identifies genes that are highly or differentially expressed in cell clusters that will be matched by a ligand-receptor database to discover important intercellular communication events.

### Data analysis and statistics

Comparisons between the two groups were made using Student’s t test and correlation coefficients were calculated using Spearman analysis. P<0.05 was considered significant. Regarding the code used in this study we have uploaded it as Supplementary File 1.

### Data availability statement

Data used in this study were obtained from Gene Expression Omnibus (GEO) database (https://www.ncbi.nlm.nih.gov/geo).

## RESULTS

### Global single-cell landscape of bone metastases from breast cancer

**Figure 1:**
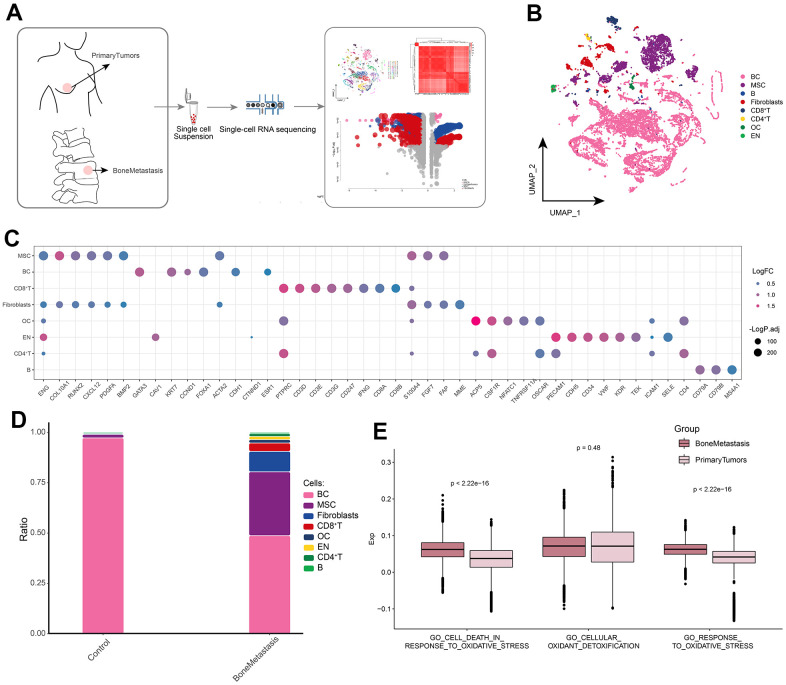
**Global single-cell landscape of patients with bone metastases from breast cancer.** (**A**) Flow chart underlying this study. Primary tumors were used as controls in the follow-up study. (**B**) Single-cell atlas mapping cell types. (**C**) Cell marker genes for annotation. (**D**) Differences in cell abundance between control and breast cancer bone metastasis patients. (**E**) Comparison of differences in single-cell oxidative stress levels between control and bone metastasis samples.

We try to draw the global single cell atlas of BM through scRNA-seq technology, and further explore the potential ecological panorama of BM, in order to find the potential therapeutic target of BM. The flow of this study was shown in Figure 1A. After standardized data processing and quality control, a total of 16,409 high-quality single-cell transcriptional profiles were captured and clustered to generate 43 cell clusters, and differential gene expression analysis revealed a wide range of gene expression dysregulation in different kinds of cell types in BM compared to controls. The cell clusters were further identified into eight cell types (Figure 1B), including BC cells, osteoblasts (OC), mesenchymal stem cells (MSCs), B cells, CD8^+^ T cells, CD4^+^ T cells, fibroblasts and endothelial cells (En). The markers positively expressed by the cells are consistent with recent published gene signatures such as scRNA-seq and laboratory studies (Figure 1C). In addition, BC cells were reduced in the BM microenvironment compared to controls, whereas MSCs, Fibroblasts and other cells were significantly more abundant in the BM (Figure 1D). In addition, we assessed the differences in single-cell oxidative stress levels between control and BM samples and found that GO_CELL_DEATH_IN_RESPONSE_TO_OXIDATIVE_STRESS and GO_RESPONSE_TO_OXIDATIVE_STRESS had higher scores in bone metastasis samples (Figure 1E). In summary, we initially constructed a global landscape of the dynamic single-cell ecology of the BM microenvironment by single-cell histology, and we found significant concomitant dysregulation of gene expression between different cell types and explored the altered cellular ecology of BM patients, in which BC, MSCs and fibroblasts may play an important role in BM. In addition to this, oxidative stress may play a key role in BM.

### Landscape of BC cell subpopulations in breast cancer bone metastases

**Figure 2:**
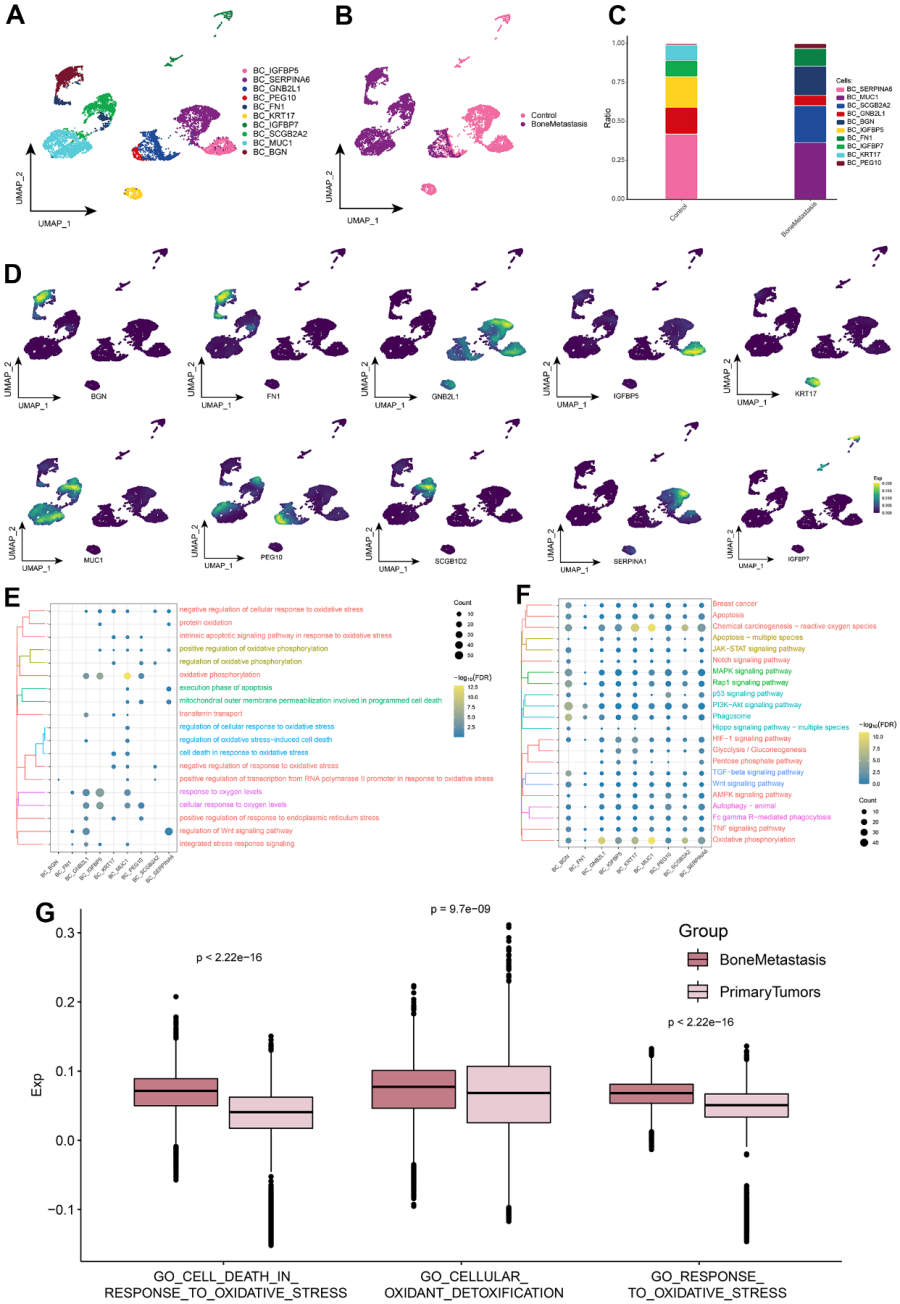
**Breast cancer cell subpopulations in patients with bone metastases from breast cancer.** (**A**) Single-cell atlas showing breast cancer cell subpopulations. (**B**) Single-cell atlas showing breast cancer cell subpopulations in control and breast cancer bone metastasis patients. (**C**) Differential abundance of breast cancer cell subpopulations in control and breast cancer bone metastasis patients. (**D**) Marker genes specifically and highly expressed in subpopulations of breast cancer cells. (**E**, **F**) Biological processes (**E**) and signaling pathways (**F**) enriched in breast cancer cell subpopulations. (**G**) Comparison of the differences in oxidative stress levels in breast cancer cells between control and bone metastasis samples.

When cancer cells metastasize to the bone, these cells enter some part of the bone through the blood or lymphatic system and become metastatic cancer cells. When these cancer cells deposit in bone, they release substances that form osteoclasts and osteoblasts. This may account for the decrease in BC cells in Figure 1D. Based on the cellular ecological atlas at single-cell resolution, we explored the subpopulations of BC cells in depth and identified a total of 10 subpopulations of BC cells (Figure 2A) and found that these subpopulations were heterogeneous among different subgroups (Figure 2B). These BC cell subpopulations all significantly expressed their marker genes, with BC_MUC1, BC_SCGB2A2, BC_FN1, BC_BGN, and BC_PEG10 subpopulations being significantly more abundant in BM (Figure 2C, 2D). By enrichment analysis, we found that these subpopulations were significantly enriched in oxidative stress response and BPs associated with oxidative stress and cell death (Figure 2E). In addition, Oxidative phosphorylation, HIF-1 signaling pathway, TGF-beta signaling pathway, Wnt signaling pathway, Apoptosis-associated oxidative stress and cell death-related pathways, MAPK signaling pathway, p53 signaling pathway, PI3K-Akt signaling pathway Breast cancer, and JAK-STAT signaling pathway of cancer-related signaling pathways were also significantly enriched (Figure 2F). Meanwhile, in control and BM samples, BC cells were significantly different from GO_CELL_DEATH_IN_RESPONSE_TO_OXIDATIVE_STRESS, GO_CELLULAR_OXIDANT_DETOXIFICATION and GO_RESPONSE_TO_OXIDATIVE_ STRESS oxidative stress pathway scores were significantly different (Figure 2G and Supplementary Figure 1). Oxidative stress-related pathway scores were significantly higher in BM samples than in controls. These results further suggest that BC cells in BM are susceptible to oxidative damage and exhibit high levels of oxidative stress, which plays a key role in apoptosis.

### Clonal evolution of BC cells in bone metastases from breast cancer

**Figure 3:**
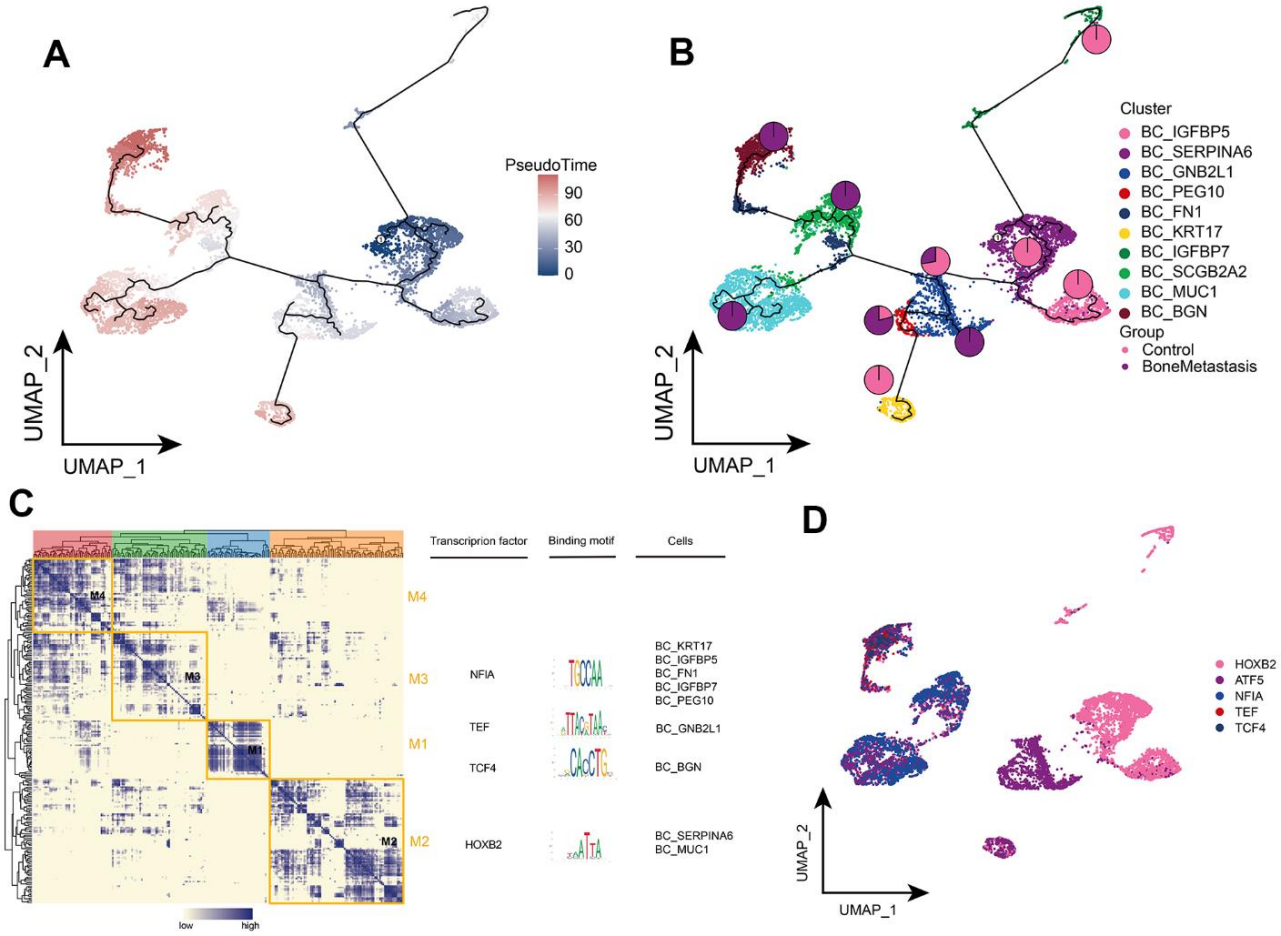
**Clonal evolution of breast cancer cell subpopulations in patients with bone metastases from breast cancer.** (**A**, **B**) Pseudo-time values (**A**) and developmental trajectories (**B**) of breast cancer cell subpopulations, with pie charts representing the proportion of control and breast cancer bone metastasis patients in breast cancer cell subpopulations. (**C**) Co-expression modules of transcription factors in breast cancer cell subpopulations of patients with breast cancer bone metastases. Left: Identification of regulator modules based on the regulator’s linkage specificity index matrix. Middle: representative transcription factors and their binding patterns in the modules. Right panel: cell subpopulations in which transcription factors are located. (**D**) Single-cell atlas showing transcription factors regulating breast cancer cell subpopulations.

We then depicted the differentiated developmental trajectories of tumor cell subpopulations. BC_MUC1, BC_SCGB2A2, and BC_BGN subpopulations were at the end of the developmental trajectory of tumor cells, while BC_SERPIINA6 subpopulation was at the beginning of the developmental trajectory and gradually differentiated into different other subpopulations as BM progressed (Figure 3A, 3B). Markers were clustered into four modules, where BC_MUC1 and BC_SERPIINA6 were regulated by the same transcription factor HOXB2 and BC_BGN was regulated by the TCF4 transcription factor (Figure 3C), and the expression of these transcription factors was mapped in a single cell atlas (Figure 3D). In conclusion, we further clarified the differentiation and development trajectory of tumor cell subsets in BM and the transcriptional regulation targets of different subsets.

### Landscape of fibroblast subpopulations in bone metastases from breast cancer

**Figure 4:**
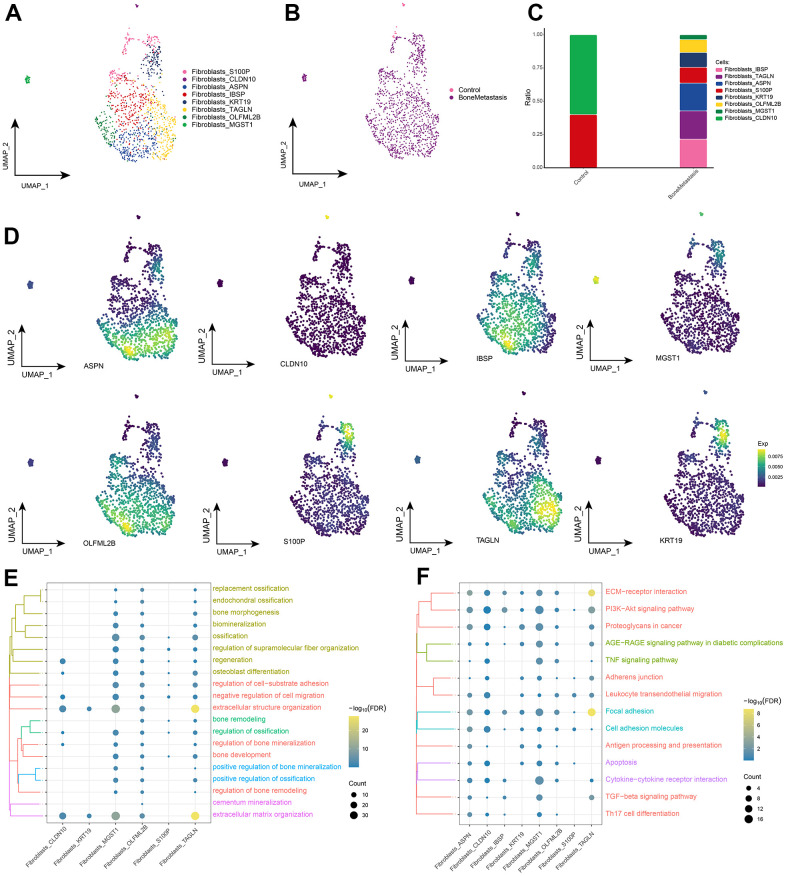
**Fibroblast subpopulations in patients with bone metastases from breast cancer.** (**A**) Single-cell atlas showing fibroblast subpopulations. (**B**) Single-cell atlas showing fibroblast subpopulations in control and breast cancer bone metastasis patients. (**C**) Differences in abundance of fibroblast subpopulations in control and breast cancer bone metastasis patients. (**D**) Marker genes specifically and highly expressed in subpopulations of fibroblast subpopulations. (**E**, **F**). Biological processes (**E**) and signaling pathways (**F**) that enrich fibroblast subpopulations.

The aforementioned of others’ analysis revealed the presence of a large number of fibroblasts in bone metastasis sites, and fibroblasts are becoming important cellular players in bone metastasis [21]. Eight fibroblast subpopulations were further identified by subpopulation analysis (Figure 4A), and these fibroblast subpopulations were essentially highly enriched in BM (Figure 4B), with differentially expressed genes for each subpopulation displayed in Supplementary Table 1. These fibroblast subpopulations all expressed different specific markers, and studies of their variable levels revealed significant abundance of Fibroblasts_CLDN10 and Fibroblasts_S100P in controls, and Fibroblasts_IBSP, Fibroblasts_TAGLN, Fibroblasts_ASPN, and Fibroblasts_OLFML2B, Fibroblasts_KRT19 and Fibroblasts_MGST1 were significantly abundant in BM (Figure 4C, 4D). Further, the BPs and KEGG signaling pathways involved in these fibroblast subpopulations were explored, and fibroblast subpopulations were found to be significantly involved in the biological processes of ossification and bone remodeling (Figure 4E), in addition to ECM-receptor interaction, Cytokine-cytokine receptor interaction, Apoptosis, Focal adhesion, TNF signaling pathway, and TGF-beta signaling pathway were significantly enriched (Figure 4F). Taken together, our results suggest that fibroblasts in BM may regulate the ecological niche formation of bone metastases and play an important role for tumor cell inoculation into the bone marrow and growth.

### Clonal evolution of fibroblasts in bone metastases from breast cancer

**Figure 5:**
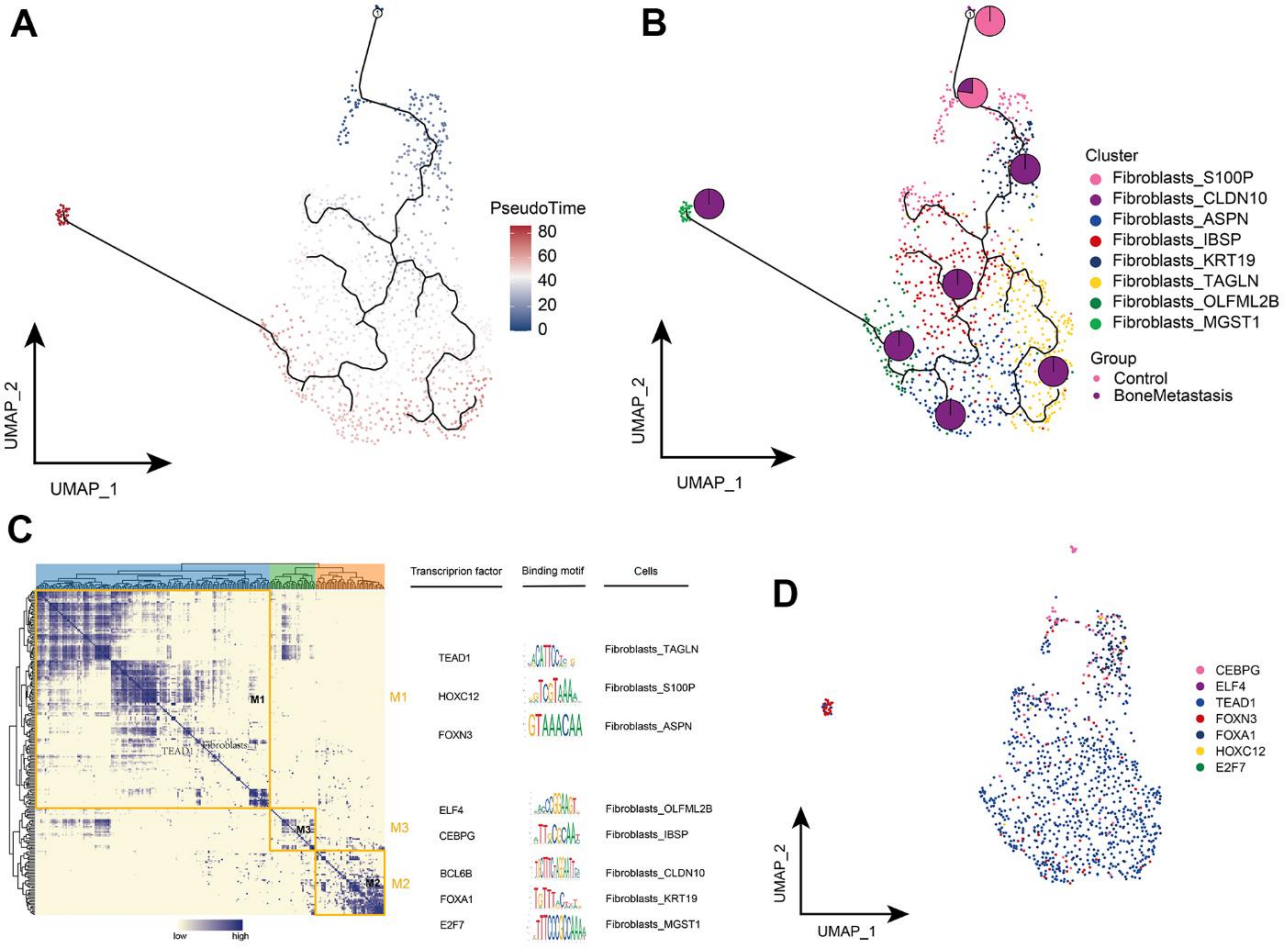
**Clonal evolution of fibroblast subpopulations in patients with bone metastases from breast cancer.** (**A**, **B**) Pseudo-time values (**A**) and developmental trajectories (**B**) of fibroblast subpopulations, with pie charts representing the proportion of fibroblast subpopulations in control and breast cancer bone metastasis patients. (**C**) Co-expression modules of transcription factors in fibroblast subpopulations of patients with breast cancer bone metastases. Left: Identification of regulator modules based on the regulator**’**s linkage specificity index matrix. Middle: representative transcription factors and their binding patterns in the modules. Right panel: cell subpopulations in which transcription factors are located. (**D**) Single-cell atlas showing transcription factors regulating fibroblast subpopulations.

The pseudo-time differentiation trajectory showed that the Fibroblasts_S100P and Fibroblasts_CLDN10 subpopulations were in an early position of differentiation development, while Fibroblasts_IBSP, Fibroblasts_TAGLN, Fibroblasts_ASPN, Fibroblasts_ OLFML2B, Fibroblasts_KRT19, and Fibroblasts_MGST1 were at the end stage and all were highly enriched in BM (Figure 5A, 5B). Subsequent GRN analysis showed that fibroblast subpopulation genes were organized into three modules (Figure 5C) and that different fibroblast subpopulations were regulated by different TFs to guide cell fate selection, respectively (Figure 5D). These results reflect the developmental trajectory of fibroblast subpopulations in BM and the transcriptional regulatory targets of different subpopulations.

### The MSC subpopulations landscape of breast cancer bone metastases

**Figure 6:**
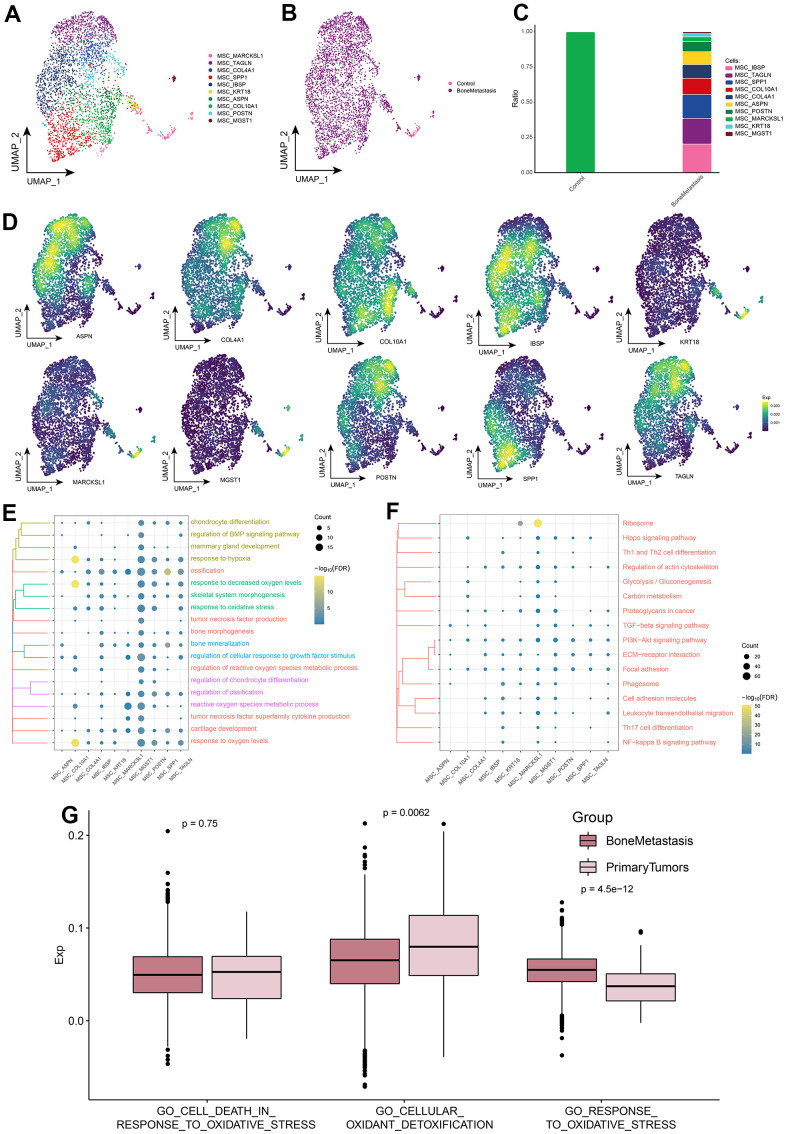
**MSC subpopulations in patients with bone metastases from breast cancer.** (**A**) Single-cell atlas showing MSC subpopulations. (**B**) Single-cell atlas showing MSC subpopulations in control and breast cancer bone metastasis patients. (**C**) Differential abundance of MSC subpopulations in control and breast cancer bone metastasis patients. (**D**) Marker genes specifically and highly expressed in subpopulations of MSC subpopulations. (**E**, **F**) Biological processes (**E**) and signaling pathways (**F**) enriched in MSC subpopulations. (**G**) Comparison of the differences in oxidative stress levels in MSC between control and bone metastasis samples.

MSCs can differentiate into a variety of cell types, including osteogenic osteoblasts, chondrocytes, and adipocytes [22]. In addition, MSCs are a key component of tumor cell homing and adhesion to bone metastasis ecological niches [2]. Exploring the MSC subpopulation landscape by single cell resolution identified ten MSC subpopulations (Figure 6A), all of which were essentially significantly abundant in BM (Figure 6B), with differentially expressed genes for each subpopulation displayed in Supplementary Table 2. Further exploring the expression of specific markers for these subpopulations and differences in the abundance of subpopulations, the MSC_MARCKSL1 subpopulation was significantly more abundant in the control group, while all other subpopulations were significantly present in the BM group (Figure 6C, 6D). Enrichment analysis revealed that MSC subpopulations were involved in the regulation of BMP signaling pathway, tumor necrosis factor production, tumor necrosis factor superfamily cytokine production, and some skeletal growth and development and oxidative stress-related BPs (Figure 6E), Focal adhesion, Cell adhesion molecules, ECM-receptor interaction, which are KEGG signaling pathways associated with tumor metastasis, were enriched, in addition, PI3K-Akt signaling pathway, TGF-beta signaling pathway, Hippo signaling pathway and NF-kappa B signaling pathway, which are cancer-related KEGG signaling pathways, were also significantly enriched (Figure 6F). GO_RESPONSE_TO_OXIDATIVE_ STRESS scores were higher in the BM samples (Figure 6G and Supplementary Figure 2). In conclusion, by identifying MSC subpopulations and uncovering the functional roles of their subpopulations, we found that MSCs and oxidative stress may play an important role in BM.

### Clonal evolution of MSCs with bone metastases from breast cancer

**Figure 7:**
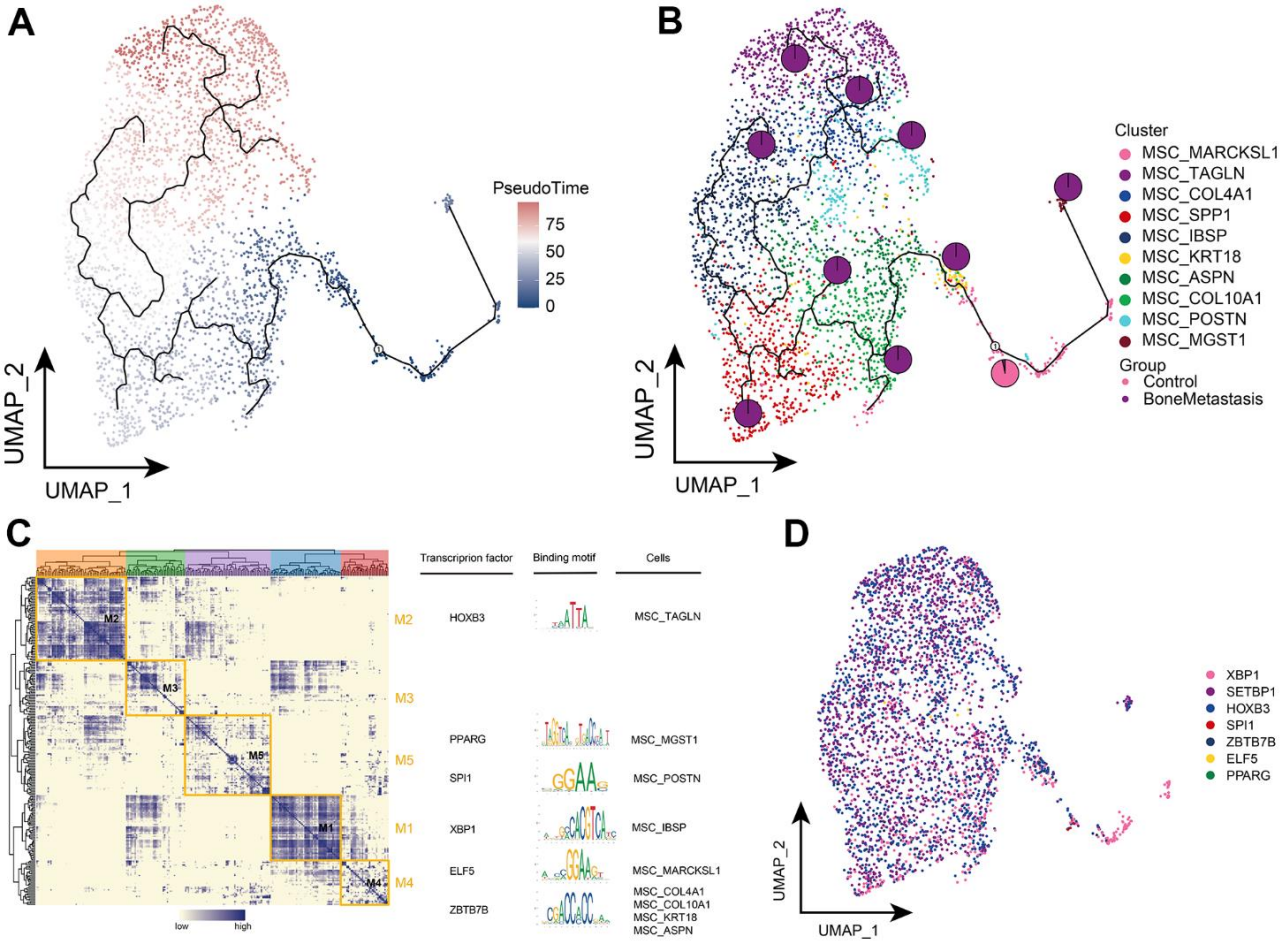
**Clonal evolution of MSC subpopulations in patients with bone metastases from breast cancer.** (**A**, **B**) Pseudo-time values (**A**) and developmental trajectories (**B**) of MSC subpopulations, pie charts representing the proportion of control and breast cancer bone metastasis patients in MSC subpopulations. (**C**) Co-expression modules of transcription factors in MSC subpopulations of patients with breast cancer bone metastases. Left: Identification of regulator modules based on the regulator**’**s linkage specificity index matrix. Middle: representative transcription factors and their binding patterns in the modules. Right panel: cellular subpopulations in which transcription factors are located. (**D**) Single-cell atlas showing transcription factors regulating MSC subpopulations.

Further exploring the differentiation trajectory of the MSC subpopulations, the MSC_MARCKSL1 subpopulation was located at an early position in the development of differentiation and had a high ability to differentiate further toward subpopulations specifically present in the BM (Figure 7A, 7B). Confirming the accuracy of the results in Figure 6C, the MSC_MARCKSL1 subpopulation was significantly more abundant in the primary lesions. By GRN, we explored the TFs regulating MSC subpopulations that are regulated by different TFs, including HOXB3, PPARG, SPI1, XBP1, ELF5, and ZBTB7B, respectively (Figure 7C, 7D). Taken together, we can know the differentiated developmental trajectory of MSC subpopulations and explored their transcriptional regulation.

### Intercellular communication in breast cancer bone metastases

**Figure 8:**
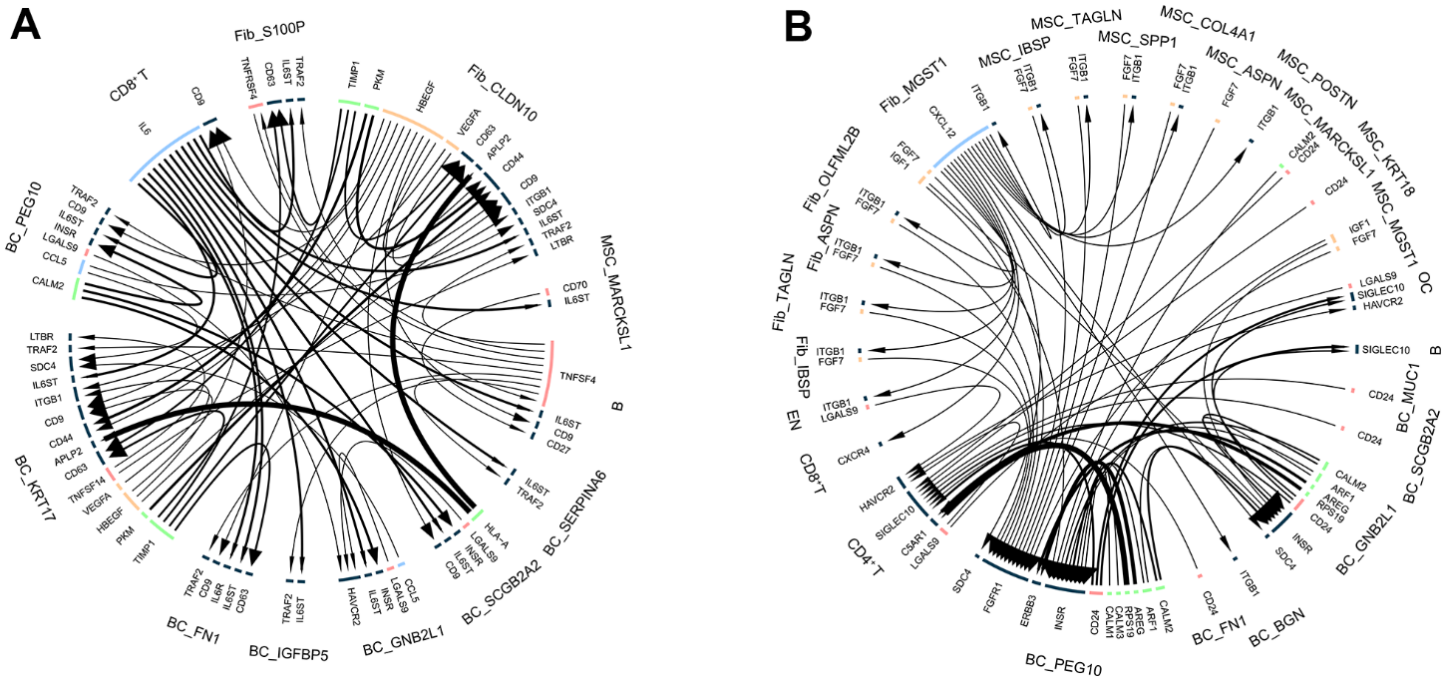
**Intercellular communication in breast cancer bone metastases.** (**A**) Intercellular communication of cell subpopulations in control samples. (**B**) Intercellular communication of cell subpopulations in bone metastasis samples.

Since we have successfully outlined cellular-level alterations in BM and characterized the functional and transcriptional profiles of different cellular subpopulations, we used a public ligand-receptor database to infer intercellular communication during BM. By comparing cell identity-specific genes with ligand receptors, we classified hypothetical ligand-receptor pairs for different cell populations in control and BM samples. For controls, we found a stronger interaction between fibroblasts and BC cells (Figure 8A). Notably, in BM, BC cells showed the most interactions with other cell types (Figure 8B and Supplementary Table 3), and we observed strong ligand-receptor pairs in BC cell subpopulations.

## DISCUSSION

Recurrence of ER+ breast cancer leads to high mortality rates every year, therefore the specificity of BM must be identified by dissecting the mechanism of action between single cells. The development of bone metastases in breast cancer patients is characterized by complications and poor prognosis, as well as leading to a reduced quality of life for patients. In BM, different cells have different roles in cancer progression and metastasis, and some cells with significant differences are emerging as important cellular players in BM [21, 22]. Here, we constructed a global single-cell landscape of BM based on scRNA-seq data from primary tumor tissues of BM patients and BM tissues, and further explored the pathways, differentiation developmental trajectories and transcriptional regulatory targets involved in cell subpopulations that may have important roles in BM.

Bone is the preferred site of BC cells metastasis, and after metastasis to bone, tumor cells regulate the interactions between different cell types through molecular mechanisms that alter bone homeostasis and thus tumor cell survival, dormancy and/or proliferation [23]. In the present study, tumor cells were observed to be reduced in BM and highly expressed MUC1, SCGB2A2, FN1, BGN, and PEG10. Mucin 1 (MUC1), also known as cancer antigen 15-3 (CA 15-3), has the potential to promote BC cell motility and metastasis. It has been demonstrated that MUC1 adheres to E-selectin and intercellular adhesion molecule-1 (ICAM-1) on the endothelial surface and that ICAM-1 activates the Src oncogene, thereby enhancing BC cell motility and metastatic potential [24]. A study by Iman Mamdouh Talaat et al. tentatively demonstrated that bone marrow horse injection hemoglobin-1 (SCGB2A2) can be used as a tool to study breast cancer early BM. Fibronectin 1 (FN1) is an extracellular matrix protein that may play an important role in inhibiting BC-associated bone loss. In contrast, BGN and PEG10 have not been studied in BM. In addition, enrichment analysis revealed that breast cancer cells in BM are susceptible to oxidative damage and exhibit high levels of oxidative stress, which plays a key role in apoptosis. The mechanistic role of the oxidative microenvironment on BC cells in bone remains largely controversial [25, 26]. Previous studies have shown that in breast cancer, oxidative stress has different effects on primary tumors and distal metastatic organs at different pathological stages [27]. However, recent studies have found that accumulation of oxidative stress may lead to tumor cell death [28]. This is consistent with our study.

To date, only preliminary studies have been performed on the phenotype and transcript levels of fibroblasts in patients with bone metastases. BM often proceeds through multiple steps, including multiple metastases from the primary site of cancer cell growth, invasion, migration through the body circulation and extravasation, seeding to distant organs and subsequent steps of proliferation therein [29, 30]. Subpopulation analysis revealed significant abundance of Fibroblasts_CLDN10 and Fibroblasts_S100P in controls, Fibroblasts_IBSP, Fibroblasts_TAGLN, Fibroblasts_ASPN, Fibroblasts_OLFML2B, Fibroblasts Fibroblasts_KRT19 and Fibroblasts_MGST1 were significantly abundant in BM. analysis by Jinling Liao et al. revealed that CLDN10 expression levels were reduced in breast cancer tissues compared to normal breast tissues [31]. In addition, a pro-metastatic and developmental role of S100P in BC has been identified. IBSP attracts osteoclasts and creates an osteoclast-rich environment in bone, assisting in the delivery of exosomal miR-19a to osteoclasts to induce osteoclastogenesis [32]. And TAGLN, ASPN, KRT19 and MGST1 have important roles in the prognosis, invasion, metastasis and drug resistance of breast cancer [33–36]. In the present study, fibroblast subpopulations were found to be significantly involved in Cytokine-cytokine receptor interaction, TNF signaling pathway and TGF-beta signaling pathway, and Focal adhesion, and fibroblasts can contact in an intercellular dependence to regulate migration and invasion capacity. Force transmission is mediated by heterogeneous adhesion involving N-calmodulin on fibroblast membranes and E-calmodulin on cancer cell membranes. Fibroblast-derived cytokines and chemokines can contribute to the immunosuppressive tumor microenvironment by recruiting and producing immunosuppressive cells [21]. These results suggest a role of fibroblast subpopulations in the promotion of BM.

During the BM process, BC cells migrate together with MSCs from the primary foci to the bone marrow, a process that is dependent on bone bridge proteins [37]. Interestingly, the MSC_MARCKSL1 subpopulation was found in this study to be located at an early position in differentiation development, with a high differentiation capacity to further differentiate to subpopulations specifically present in BM. In addition, the prognostic value of MARCKSL1 in breast cancer has been gradually investigated [38, 39]. However, its regulatory role in BM is still unknown, and this study proposes to imagine MARCKSL1 as an important regulatory molecule in the BM process, and its study may provide help for the treatment of BM. In addition, MSCs have a strong osteogenic potential [22], and in the present study it was also found that MSC subpopulations were significantly enriched in some BPs related to skeletal growth and development and oxidative stress. Compared to highly differentiated cell types, MSCs have a weaker antioxidant capacity and is more sensitive to oxidative responses [40]. These studies highlight the complexity of MSCs and further studies are needed to understand whether MSCs can be used clinically in the treatment of bone metastases.

Previous studies focused on the exploration of a single gene or a unique type of cell in BM. However, no comprehensive single cell profiling analysis of BM has been conducted, largely ignoring the impact of intercellular interactions on BM. In conclusion, our results provide a preliminary subpopulation landscape of the BM tumor microenvironment and reveal the differentiated developmental trajectories and transcriptional regulatory targets of these subpopulations, revealing the transcriptional heterogeneity hidden in the population-averaged measurements and providing ideas for identifying new targets for personalized therapeutic approaches. However, the results need to be validated using experiments and larger samples.

## Supplementary Material

Supplementary File 1

Supplementary Figures

Supplementary Table 1

Supplementary Table 2

Supplementary Table 3
