# Supplementary material for: Single-cell sequencing analysis reveals the relationship between tumor microenvironment cells and oxidative stress in breast cancer bone metastases
Source: Aging (Albany NY). 2023 Jul 19;15(14):6950–68. doi: 10.18632/aging.204885 (PMC10415571; doi:10.18632/aging.204885)
Supplement: Supplementary File 1 [file aging-15-204885-s001.docx]

Supplementary File 1. The R code used in this study

# scCode

library(Seurat)

library(tidyverse)

options(stringsAsFactors = F)

mycol <- c("#D20A13","#223D6C","#FFD121","#91612D","#08FCFC","#11AA4D","#58CDD9","#7A142C","#4300FF","#5D90BA","#431A3D","#088247","#6E568C","#E0367A","#D8D155","#64495D","#7CC767")

expression_matrix <-Read10X("./")

seurat_obj <- CreateSeuratObject(count = expression_matrix,

min.cells = 3,

min.features = 200)

#

seurat_obj[['percent.mt']] <- PercentageFeatureSet(seurat_obj, pattern = "^MT-")

pdf("1.VlnPlot.pdf")

VlnPlot(seurat_obj, features = c("nFeature_RNA", "nCount_RNA", "percent.mt"), ncol = 3)

dev.off()

seurat_obj <- subset(seurat_obj, subset = nFeature_RNA > 200 & nFeature_RNA < 5000 & percent.mt < 30)

#

seurat_obj <- NormalizeData(seurat_obj,

normalization.method = "LogNormalize",

scale.factor = 10000)

# HVG(highly variable features)

seurat_obj <- FindVariableFeatures(seurat_obj,selection.method = "vst",

nfeatures = 2000,num.bin = 20)

#

all.genes <- rownames(seurat_obj)

seurat_obj <- ScaleData(seurat_obj, features = all.genes)

# PCA

seurat_obj <- RunPCA(seurat_obj,

features = NULL,

npcs = 100,

ndims.print = 1:5,

nfeatures.print = 5)

#

pdf("2.VizDimLoadings.plot.pdf")

VizDimLoadings(seurat_obj, dims = 1:2, reduction = "pca")

dev.off()

pdf("3.DimPlot.pdf")

DimPlot(seurat_obj, reduction = "pca")

dev.off()

pdf("4.DimHeatmap.pdf")

DimHeatmap(seurat_obj, dims = 1, cells = 500, balanced = TRUE)

dev.off()

pdf("5.ElbowPlot.pdf")

ElbowPlot(seurat_obj)

dev.off()

#

seurat_obj <- FindNeighbors(seurat_obj, dims = 1:50)

seurat_obj <- FindClusters(seurat_obj, resolution = 0.5)

#

levels(seurat_obj)

# save(seurat_obj,file = "seurat_obj.rdata")

#

library(ggplot2)

seurat_obj <- Run(seurat_obj,dims = 1:50,n.neighbors=30,min.dist=0.3)

pdf("6.DimPlot_cluster.pdf",width = 7,height = 6)

DimPlot(seurat_obj,

reduction = "",

pt.size = 0.2,

label = TRUE) +

theme(panel.border = element_blank(),axis.line = element_line(colour = "black",size=1),

panel.grid.major = element_blank(),

panel.grid.minor = element_blank())

dev.off()

save(seurat_obj,file = "seurat_obj.RData")

# Anno

seurat_obj_DimRe = readRDS("./TempData/seurat_obj_DimRe.rds")

Color = readRDS("./TempData/Color.rds")

Size = read.table("./TempData/Size.txt",sep = "\t",header = T,quote = "")

Width = read.table("./TempData/Width.txt",sep="\t",header = T)

Height = read.table("./TempData/Height.txt",sep="\t",header = T)

s_meta <-seurat_obj_DimRe@meta.data

CellType = read.table("./TempData/CellType.txt",sep="\t",header = T)

if("CellType" %in% colnames(s_meta)){

ColName = data.frame(ColName = colnames(s_meta))

ColName_1 = data.frame(ColName,Num = rownames(ColName))

NumCellType = as.numeric(ColName_1[ColName_1$ColName == "CellType",2])

colnames(s_meta)[NumCellType] = "CellType"

}

s_meta2 = merge(s_meta,CellType,by.x="seurat_clusters",by.y="Cluster",all.x = T,sort=F)

rownames(s_meta2) <-s_meta2$cells

s_meta3 = s_meta2[rownames(s_meta),]

seurat_obj_DimRe@meta.data <- s_meta3

names(seurat_obj_DimRe@meta.data)

cell2type <-data.frame(cells=pred.cells@rownames,pred.cells=pred.cells$labels)

meta <-seurat_obj@meta.data

meta$cells <-rownames(meta)

meta2 <-merge(meta,cell2type,by = "cells",sort = F)

rownames(meta2) <-meta2$cells

seurat_obj@meta.data <- meta2

names(seurat_obj@meta.data)

Idents(seurat_obj) <- "pred.cells"

mycol <- c("#D20A13","#223D6C","#FFD121","#91612D","#08FCFC","#11AA4D","#58CDD9","#7A142C","#4300FF","#5D90BA","#431A3D","#088247","#6E568C","#E0367A","#D8D155","#64495D","#7CC767")

pdf("6.DimPlot.pdf",width = 7,height = 6)

DimPlot(seurat_obj,

reduction = "",

pt.size = 0.1,

label = T,

cols = colorRampPalette(mycol)(16)) +

ggtitle("RefAML") +

theme(panel.border = element_blank(),axis.line = element_line(colour = "black",size=1),

panel.grid.major = element_blank(),

panel.grid.minor = element_blank(),

plot.title = element_text(hjust = 0.5))

dev.off()

cellTypes <-data.frame(table(cellType=pred.cells$labels,cluster=seurat_obj$RNA_snn_res.0.5))

cellTypes <-cellTypes[cellTypes$Freq>0,]

cellTypes <-spread(data=cellTypes,cluster,value = Freq)

cellTypes[is.na(cellTypes)]<-0

write.csv(cellTypes,file = "cellTypes.csv",row.names = F,quote = F)

s_meta <-seurat_obj@meta.data

s_meta$cells <-rownames(s_meta)

s_meta$seurat_clusters <-as.character(s_meta$seurat_clusters)

seurat_obj@meta.data <- s_meta

names(seurat_obj@meta.data)

Idents(seurat_obj) <- "seurat_clusters"

stack_color <-c("#1177BB","#FF7700","#229966","#DD2222","#AA44FF","#885544","#EE77CC","#BBBB66","#11BBCC","#AACCEE","#FFBB77","#87ED88","#FF9999","#CCBBDD","#CC9999","#FFBBDD","#DDDD88","#99DDEE","#AA4444","#886633")

pdf("6.DimPlot_final.pdf",width = 8,height = 8)

DimPlot(seurat_obj,

reduction = "",

pt.size = 0.1,

label = F,

cols = stack_color ) +

ggtitle("DimPlot") +

theme(panel.border = element_blank(),axis.line = element_line(colour = "black",size=1), # 去除默认填充的灰色，并将x=0轴和y=0轴加粗显示(size=1)

panel.grid.major = element_blank(),

panel.grid.minor = element_blank(),

plot.title = element_text(hjust = 0.5)) +

coord_fixed(1)

dev.off()

seurat_obj_final <- seurat_obj

save(seurat_obj_final,file = "seurat_obj_final.rdata")

load("data_stemness.rdata")

meta <-seurat_obj_final@meta.data

ans <-merge(meta,data_stemness,by.x="cells",by.y="Sample")

ans <-ans[,c("seurat_clusters","StemnessScore")]

medianStem <-ans %>% group_by(seurat_clusters) %>% summarise(median=median(StemnessScore))

#

library(dplyr)

final_cluster <- levels(seurat_obj_final)

# all markers

markers_all <- FindAllMarkers(seurat_obj_final, only.pos = FALSE, min.pct = 0.25, logfc.thresh = 0.25)

write.table(markers_all, "markers_all.txt",row.names = F,quote = F,sep="\t")

markers_ROC_all <- FindAllMarkers(seurat_obj_final, only.pos = FALSE, test.use = "roc", min.pct = 0.25, logfc.thresh = 0.25)

write.table(markers_ROC_all, "markers_ROC_all.txt",row.names = F,quote = F,sep="\t")

#

markers_pos <- FindAllMarkers(seurat_obj_final, only.pos = TRUE, min.pct = 0.25, logfc.thresh = 0.25)

write.table(markers_pos, "markers_pos.txt",row.names = F,quote = F,sep="\t")

markers_ROC_pos <- FindAllMarkers(seurat_obj_final, only.pos = TRUE, test.use = "roc", min.pct = 0.25, logfc.thresh = 0.25)

write.table(markers_ROC_pos, "markers_ROC_pos.txt",row.names = F,quote = F,sep="\t")

#

fcolor <-c("#134479","#2E76B1","#9ECAE0","#E0ECF2","white","#F8EAE0","#F5BDA3","#E07964","#A1132B")

expression.color <- c("darkblue", "lightblue", "green", "yellow", "red")

min <-seurat_obj_final@assays$RNA@data["PTPRC",] %>% range %>% .[1]

max <-seurat_obj_final@assays$RNA@data["PTPRC",] %>% range %>% .[2] %>% round(.,2)

#fcolor <-c("#134479","#71ADD0","#8ABFD9","#D0E3ED","gray99","#F8EEE7","#F9E9DF","#FBDCCB","#F6BDA3","#F1A384","#E99076","#BC323A","#7A0824")

#fcolor <-c("#134479","#2E76B1","#9ECAE0","#F7F5F4","#F5BDA3","#E07964","#A1132B")

pdf("8.FeaturePlot.pdf",width = 6,height = 6)

FeaturePlot(seurat_obj_final,

features ="PTPRC",

pt.size = 0.2,

cols = c("gray90","red"),

label = FALSE,

label.size = 4) +

scale_color_gradientn(colors = expression.color)

dev.off()

#

adj_DoHeatmap <-function(obj,features){

return(DoHeatmap(obj,

features = features,

size = 2,

hjust = 1.5,

angle = 0,

label = TRUE,

draw.lines = TRUE,

lines.width = 20,

group.bar.height = 0.04,

group.colors = colorRampPalette(mycol)(20)

) + NoLegend() +

scale_fill_gradient2(low = "#1C61A6", high = "#EBA28C", mid = "#273C65"))

}

pdf("9.DoHeatmap_ROC_Top1.pdf",width = 10,height = 10)

adj_DoHeatmap(seurat_obj_final,Show_markers$gene)

dev.off()

library(Seurat)

library(ggplot2)

library(monocle3)

mycol <- c("#D20A13","#223D6C","#FFD121","#088247","#11AA4D","#58CDD9","#D8D155","#5D90BA","#431A3D","#91612D","#6E568C","#E0367A","#7A142C","#64495D","#7CC767")

load("seurat_obj_final.Rdata")

metadata <-seurat_obj_final@meta.data

cells1 <-metadata$cells

gene_annotation <- as.data.frame(rownames(seurat_obj_final@reductions[["pca"]]@feature.loadings),

row.names = rownames(seurat_obj_final@reductions[["pca"]]@feature.loadings))

colnames(gene_annotation) <- "gene_short_name"

cell_metadata <- as.data.frame(cells1,

row.names = cells1)

colnames(cell_metadata) <- "barcode"

New_matrix <- seurat_obj_final@assays$RNA@counts

New_matrix <- New_matrix[rownames(seurat_obj_final@reductions[["pca"]]@feature.loadings),cells1]

expression_matrix <- New_matrix

cds_from_seurat <- new_cell_data_set(expression_matrix,

cell_metadata = cell_metadata,

gene_metadata = gene_annotation)

cds_from_seurat@metadata

# tmp.cds <- preprocess_cds(cds_from_seurat,method = "PCA",num_dim = 100)

# tmp.cds <- reduce_dimension(tmp.cds,preprocess_method = "PCA")

# tmp.cds <- cluster_cells(tmp.cds)

# tmp.cds@clusters@listData$$partitions

cds_from_seurat@clusters@listData[[""]][["clusters"]] <- Idents(seurat_obj_final)

cata(cds_from_seurat)$celltype <- seurat_obj_final@meta.data[rownames(cata(cds_from_seurat)),"seurat_clusters"]

# partitions

cds_from_seurat@clusters@listData[[""]][["partitions"]] <- "1"

#

cds_from_seurat@clusters@listData[[""]][["louvain_res"]] <- "NA"

#

cds_from_seurat@int_cata@listData$reducedDims[[""]] <-seurat_obj_final@reductions[[""]]@cell.embeddings[cells1,]

colnames(cds_from_seurat@int_cata@listData$reducedDims[[""]]) <-c("V1","V2")

# order_cells

#

cds_from_seurat@preprocess_aux$gene_loadings <- seurat_obj_final@reductions[["pca"]]@feature.loadings

cds_from_seurat@int_cata@listData$reducedDims$PCA <- seurat_obj_final@reductions[["pca"]]@cell.embeddings[cells1,]

#

cds_from_seurat_2d <- learn_graph(cds_from_seurat)

get_earliest_principal_node <- function(cds, time_bin="Prog"){

cell_ids <- which(cata(cds)[, "celltype"] == time_bin)

closest_vertex <-

cds@principal_graph_aux[[""]]$pr_graph_cell_proj_closest_vertex

closest_vertex <- as.matrix(closest_vertex[colnames(cds), ])

root_pr_nodes <-

igraph::V(principal_graph(cds)[[""]])$name[as.numeric(names

(which.max(table(closest_vertex[cell_ids,]))))]

root_pr_nodes

}

p_node_2d_1 <-get_earliest_principal_node(cds_from_seurat_2d,time_bin = "HSC")

p_node_2d_2 <-get_earliest_principal_node(cds_from_seurat_2d,time_bin = "earlyEry")

cds_from_seurat_2d <- order_cells(cds_from_seurat_2d,root_pr_nodes=c(p_node_2d_1,p_node_2d_2))

pse <-as.data.frame(pseudotime(cds_from_seurat_2d))

#pse$`pseudotime(cds_from_seurat_2d)` <-ceiling(pse$`pseudotime(cds_from_seurat_2d)`)

save(pse,file="MM_pseudotime.rdata")

plot_cells(cds_from_seurat_2d,

color_cells_by = "cluster",

#cell_size = 2,

alpha = 0.6,

label_roots= FALSE,

label_leaves=FALSE,

graph_label_size = 2,

trajectory_graph_color = NA,

trajectory_graph_segment_size = 0.5 ,

label_cell_groups = FALSE,

group_label_size = 5,

label_branch_points=FALSE) +

# scale_colour_gradient2(low="#134479", high="#A1132B",mid = "#F7F5F4",midpoint = 15)

scale_discrete_manual("colour",values = colorRampPalette(mycol)(16))

ggsave("M1.plot_cells_pseudotime.pdf",width = 6.5,height = 6)

ggsave("M1.plot_cells_cluster.pdf",width = 6.5,height = 6)

ciliated_cds_pr_test_res <- graph_test(cds_from_seurat_2d, neighbor_graph="principal_graph")

save(ciliated_cds_pr_test_res,file = "ciliated_cds_pr_test_res.rdata")

pr_deg_ids <- row.names(subset(ciliated_cds_pr_test_res, q_value < 0.01))

gene_module_df <- find_gene_modules(cds_from_seurat_2d[pr_deg_ids,], resolution= 0.01)

write.table(gene_module_df,"gene_module_df.txt",row.names = F,quote = F,sep = "\t")
