# Supplementary material for: Single-cell sequencing analysis reveals the relationship between tumor microenvironment cells and oxidative stress in breast cancer bone metastases
Source: Aging (Albany NY). 2023 Jul 19;15(14):6950–68. doi: 10.18632/aging.204885 (PMC10415571; doi:10.18632/aging.204885)
Supplement: Supplementary Figures [file aging-15-204885-s002.pdf]

SUPPLEMENTARY FIGURES

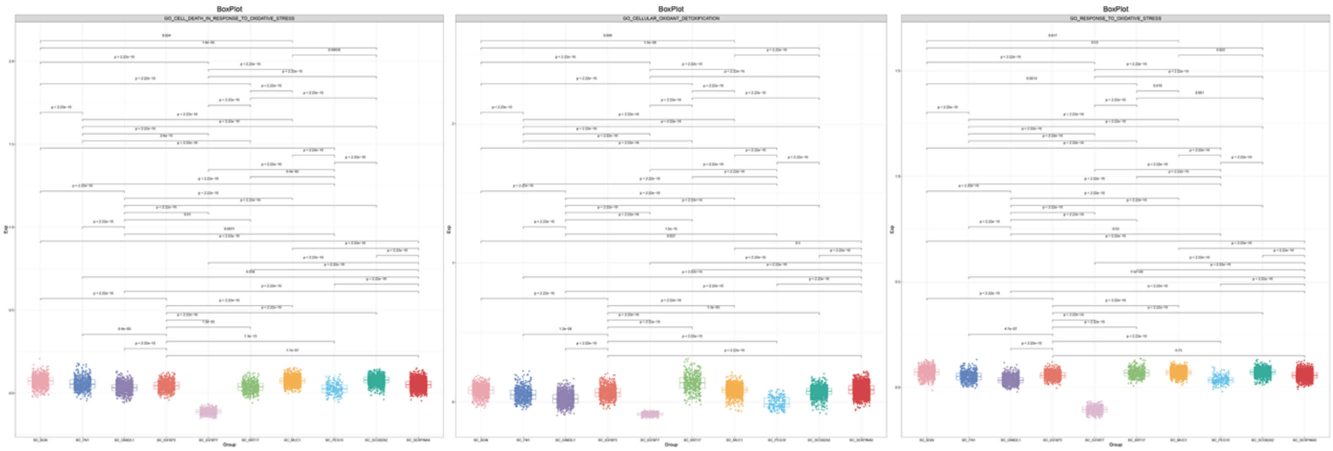

Supplementary Figure 1. Comparison of differences in oxidative stress levels in subpopulations of breast cancer cell subpopulations.

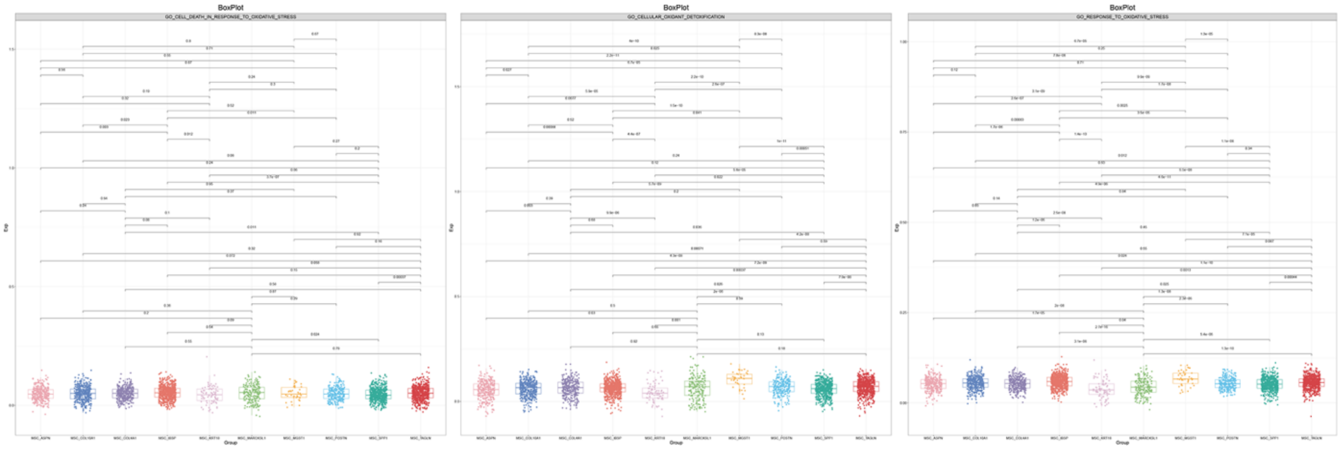

Supplementary Figure 2. Comparison of differences in oxidative stress levels in subpopulations of MSC subpopulations.
