# Supplementary material for: Single-cell sequencing analysis reveals the relationship between tumor microenvironment cells and oxidative stress in breast cancer bone metastases
Source: Aging (Albany NY). 2023 Jul 19;15(14):6950–68. doi: 10.18632/aging.204885 (PMC10415571; doi:10.18632/aging.204885)
Supplement: Supplementary Table 3 [file aging-15-204885-s005.pdf]

Supplementary Table 3. Expression of receptor ligands between cell subpopulations.

Ligand	Receptor	Cell_from_mean_exprs	Cell_from	Cell_to_mean_exprs	Cell_to	Comm_type
CALM2	INSR	19.6089743589744	BC_PEG10	20.9230769230769	BC_PEG10	other
CALM2	INSR	19.6089743589744	BC_PEG10	13.821752265861	BC_GNB2L1	other
CALM2	INSR	11.5619335347432	BC_GNB2L1	20.9230769230769	BC_PEG10	other
CALM2	INSR	11.5619335347432	BC_GNB2L1	13.821752265861	BC_GNB2L1	other
ARF1	INSR	6.46794871794872	BC_PEG10	20.9230769230769	BC_PEG10	other
AREG	ERBB3	50.2948717948718	BC_PEG10	1.91666666666667	BC_PEG10	other
RPS19	C5AR1	80.2884615384615	BC_PEG10	1.16839224513889	CD4.T	other
ARF1	INSR	6.46794871794872	BC_PEG10	13.821752265861	BC_GNB2L1	other
ARF1	INSR	4.04229607250755	BC_GNB2L1	20.9230769230769	BC_PEG10	other
CALM3	INSR	3.1474358974359	BC_PEG10	20.9230769230769	BC_PEG10	other
AREG	ERBB3	32.7009063444109	BC_GNB2L1	1.91666666666667	BC_PEG10	other
CALM1	INSR	2.91666666666667	BC_PEG10	20.9230769230769	BC_PEG10	other
ARF1	INSR	4.04229607250755	BC_GNB2L1	13.821752265861	BC_GNB2L1	other
CALM2	INSR	2.60237524234234	MSC_MARCKSL1	20.9230769230769	BC_PEG10	other
RPS19	C5AR1	45.9214501510574	BC_GNB2L1	1.16839224513889	CD4.T	other
IGF1	INSR	1.54024861578947	Fibroblasts_MGST1	20.9230769230769	BC_PEG10	growth factor
IGF1	INSR	1.54024861578947	Fibroblasts_MGST1	13.821752265861	BC_GNB2L1	growth factor
IGF1	INSR	0.960274636585366	MSC_MGST1	20.9230769230769	BC_PEG10	growth factor
FGF7	FGFR1	1.38125268624813	MSC_IBSP	10.6025641025641	BC_PEG10	growth factor
FGF7	FGFR1	1.32148095799087	Fibroblasts_ASPN	10.6025641025641	BC_PEG10	growth factor
IGF1	INSR	0.960274636585366	MSC_MGST1	13.821752265861	BC_GNB2L1	growth factor
FGF7	FGFR1	1.24366999565217	MSC_ASPN	10.6025641025641	BC_PEG10	growth factor
FGF7	FGFR1	1.23176533947368	Fibroblasts_MGST1	10.6025641025641	BC_PEG10	growth factor
FGF7	FGFR1	1.10933513627451	Fibroblasts_OLFML2B	10.6025641025641	BC_PEG10	growth factor
FGF7	FGFR1	1.07037036125	MSC_COL4A1	10.6025641025641	BC_PEG10	growth factor
FGF7	FGFR1	1.06288600401338	MSC_TAGLN	10.6025641025641	BC_PEG10	growth factor
FGF7	FGFR1	1.06204908761062	Fibroblasts_IBSP	10.6025641025641	BC_PEG10	growth factor
FGF7	FGFR1	1.0396824073741	MSC_SPP1	10.6025641025641	BC_PEG10	growth factor
FGF7	FGFR1	1.02052917982063	Fibroblasts_TAGLN	10.6025641025641	BC_PEG10	growth factor
FGF7	FGFR1	0.957525197560976	MSC_MGST1	10.6025641025641	BC_PEG10	growth factor
CD24	SIGLEC10	21.8525641025641	BC_PEG10	0.327505427777778	CD4.T	checkpoint
CXCL12	SDC4	2.60106499473684	Fibroblasts_MGST1	2.56410256410256	BC_PEG10	cytokine
CXCL12	ITGB1	2.60106499473684	Fibroblasts_MGST1	2.10531071604938	EN	cytokine
CXCL12	CXCR4	2.60106499473684	Fibroblasts_MGST1	2.09322732470862	CD8.T	cytokine
CXCL12	SDC4	2.60106499473684	Fibroblasts_MGST1	2.03021148036254	BC_GNB2L1	cytokine
CXCL12	ITGB1	2.60106499473684	Fibroblasts_MGST1	1.8766818457265	MSC_POSTN	cytokine
CXCL12	ITGB1	2.60106499473684	Fibroblasts_MGST1	1.79928626726457	Fibroblasts_TAGLN	cytokine
CXCL12	ITGB1	2.60106499473684	Fibroblasts_MGST1	1.7858228834375	MSC_COL4A1	cytokine
CXCL12	ITGB1	2.60106499473684	Fibroblasts_MGST1	1.78367909498328	MSC_TAGLN	cytokine
CXCL12	ITGB1	2.60106499473684	Fibroblasts_MGST1	1.77953439019608	Fibroblasts_OLFML2B	cytokine
CXCL12	ITGB1	2.60106499473684	Fibroblasts_MGST1	1.64979349303062	BC_BGN	cytokine
CXCL12	ITGB1	2.60106499473684	Fibroblasts_MGST1	1.64658979452055	Fibroblasts_ASPN	cytokine
CXCL12	ITGB1	2.60106499473684	Fibroblasts_MGST1	1.62992778415546	MSC_IBSP	cytokine
CXCL12	ITGB1	2.60106499473684	Fibroblasts_MGST1	1.61278043157895	Fibroblasts_MGST1	cytokine
CXCL12	ITGB1	2.60106499473684	Fibroblasts_MGST1	1.59678676223022	MSC_SPP1	cytokine
CXCL12	ITGB1	2.60106499473684	Fibroblasts_MGST1	1.59097440442478	Fibroblasts_IBSP	cytokine
CD24	SIGLEC10	10.1873111782477	BC_GNB2L1	0.327505427777778	CD4.T	checkpoint
CD24	SIGLEC10	21.8525641025641	BC_PEG10	0.051744314375	OC	checkpoint
CD24	SIGLEC10	21.8525641025641	BC_PEG10	0.0382243126984127	B	checkpoint
LGALS9	HAVCR2	0.939809984027778	CD4.T	0.854847563194444	CD4.T	checkpoint
CD24	SIGLEC10	2.05944244794979	BC_SCGB2A2	0.327505427777778	CD4.T	checkpoint
CD24	SIGLEC10	2.02310481390549	BC_MUC1	0.327505427777778	CD4.T	checkpoint
LGALS9	HAVCR2	0.694962933950617	EN	0.854847563194444	CD4.T	checkpoint
CD24	SIGLEC10	10.1873111782477	BC_GNB2L1	0.051744314375	OC	checkpoint
CD24	SIGLEC10	1.55538858648649	MSC_MARCKSL1	0.327505427777778	CD4.T	checkpoint
CD24	SIGLEC10	1.34344896457243	BC_FN1	0.327505427777778	CD4.T	checkpoint
LGALS9	HAVCR2	0.46798954875	OC	0.854847563194444	CD4.T	checkpoint
CD24	SIGLEC10	10.1873111782477	BC_GNB2L1	0.0382243126984127	B	checkpoint
CD24	SIGLEC10	0.947003539473684	MSC_KRT18	0.327505427777778	CD4.T	checkpoint
LGALS9	HAVCR2	0.939809984027778	CD4.T	0.265815408522727	OC	checkpoint
